# Physicians' intentions and use of three patient decision aids

**DOI:** 10.1186/1472-6947-7-20

**Published:** 2007-07-06

**Authors:** Ian D Graham, Jo Logan, Carol L Bennett, Justin Presseau, Annette M O'Connor, Susan L Mitchell, Jacqueline M Tetroe, Ann Cranney, Paul Hebert, Shawn D Aaron

**Affiliations:** 1 Ottawa Health Research Institute, Clinical Epidemiology Program, Ottawa, ON, Canada; 2 University of Ottawa, Faculty of Health Sciences, Ottawa, ON, Canada; 3 University of Ottawa, Faculty of Medicine, Ottawa, ON, Canada; 4 Hebrew Senior Life Institute for Aging Research and Beth Israel Deaconess Medical Center, Boston, MA, USA; 5 Division of Rheumatology, The Ottawa Hospital, Ottawa, ON, Canada; 6 Division of Respiratory Medicine, The Ottawa Hospital, Ottawa, ON, Canada

## Abstract

**Background:**

Decision aids are evidence based tools that assist patients in making informed values-based choices and supplement the patient-clinician interaction. While there is evidence to show that decision aids improve key indicators of patients' decision quality, relatively little is known about physicians' acceptance of decision aids or factors that influence their decision to use them. The purpose of this study was to describe physicians' perceptions of three decision aids, their expressed intent to use them, and their subsequent use of them.

**Methods:**

We conducted a cross-sectional survey of random samples of Canadian respirologists, family physicians, and geriatricians. Three decision aids representing a range of health decisions were evaluated. The survey elicited physicians' opinions on the characteristics of the decision aid and their willingness to use it. Physicians who indicated a strong likelihood of using the decision aid were contacted three months later regarding their actual use of the decision aid.

**Results:**

Of the 580 eligible physicians, 47% (n = 270) returned completed questionnaires. More than 85% of the respondents felt the decision aid was well developed and that it presented the essential information for decision making in an understandable, balanced, and unbiased manner. A majority of respondents (>80%) also felt that the decision aid would guide patients in a logical way, preparing them to participate in decision making and to reach a decision. Fewer physicians (<60%) felt the decision aid would improve the quality of patient visits or be easily implemented into practice and very few (27%) felt that the decision aid would save time. Physicians' intentions to use the decision aid were related to their comfort with offering it to patients, the decision aid topic, and the perceived ease of implementing it into practice. While 54% of the surveyed physicians indicated they would use the decision aid, less than a third followed through with this intention.

**Conclusion:**

Despite strong support for the format, content, and quality of patient decision aids, and physicians' stated intentions to adopt them into clinical practice, most did not use them within three months of completing the survey. There is a wide gap between intention and behaviour. Further research is required to study the determinants of this intention-behaviour gap and to develop interventions aimed at barriers to physicians' use of decision aids.

## Background

Decision aids are evidence-based tools designed to prepare patients to participate in making specific and deliberative choices among healthcare options in ways they prefer. These tools assist decision making by providing evidence-based information about a health condition, the options for treatment, associated benefits, harms, probabilities, and scientific uncertainties. Decision aids help patients to recognize the values-sensitive nature of the decision and to clarify the value they place on the benefits, harms, and scientific uncertainties. This can be done by describing the options in enough detail that patients can imagine what it is like to experience the physical, emotional, and social effects; and by guiding patients to consider which benefits and harms are most important to them. Decision aids provide structured guidance in both the steps of decision making and the communication of one's informed values with others involved in the decision (e.g. clinician, family, friends). The ultimate goal of decision aids is to improve the process of decision-making and the decision quality. They are intended to supplement, not replace, practitioner counselling.

The most recent Cochrane systematic review of evidence from randomized trials demonstrated that decision aids are superior to usual care in improving key indicators of decision quality[1]. Decision aids were superior to comparison interventions in improving: knowledge of the facts and options; realistic perceptions of outcome probabilities; and agreement between patients' values and choice. Additionally, patients who used decision aids were shown to have lower decisional conflict, participate more actively in decision-making, and be less likely to remain undecided. The review also showed that exposure to decision aids reduced elective invasive surgery in favour of conservative options by 24% without adversely effecting patients' health outcomes, satisfaction, or anxiety.

Despite the evidence that decision aids support patients in making evidence-informed choices, relatively little is known about the acceptability of decision aids to practitioners or the factors that influence their use of them with patients. Furthermore, patients may not be able to benefit from decision aids if physicians resist their use. While some studies have identified barriers to implementing decision aids in the U.S. and Canadian healthcare systems [2-4], none have used representative samples of physicians to elicit their perceptions of specific decision aids or intentions to use them. The purpose of this study was to describe physicians' perceptions of three patient decision aids, their expressed intentions to use the aids and their subsequent use of them.

## Methods

### Design

We conducted a cross-sectional survey of: a) family physicians, regarding a decision aid for menopausal women considering hormone replacement therapy (HRT) [5-7]; b) geriatricians, regarding a decision aid for individuals faced with making a decision about long-term placement of a feeding tube in a cognitively impaired older person[8]; and c) respirologists or pulmonologists, regarding a decision aid for individuals with chronic obstructive pulmonary disease (COPD) considering mechanical ventilation at the end of their lives[9]. These decision aids were purposefully selected to represent a range of decisions made in primary and tertiary care and to represent a spectrum of decisions, including lifestyle and end-of-life decisions, faced by both patients and substitute decision makers. Also, these decision aids have previously been shown to have beneficial effects on knowledge[6,8], realistic expectations[5,6,9], decisional conflict[5,6,8,9] and the proportion of patients remaining undecided about treatment choice[6,8,9]. The survey was administered following Dillman's[10] total design method for mail surveys, a method designed to enhance survey response rates. One week in advance of the first mailing a letter, signed by a clinician co-investigator who was a peer of the respondent, was sent to each of the physicians. The letter was sent to explain the purpose of the study and to alert respondents to the launch of the survey. One week later, respondents were sent the decision aid, the questionnaire, an addressed stamped envelope, and an honorarium cheque for $50 (CAD). Reminders were sent to non-respondents at 3, 5, 7, 9, and 14 weeks after the initial mailing.

Three months following completion of the mail survey, respondents who had indicated on the questionnaire that they intended to use the decision aid in their clinical practice were administered a brief follow-up telephone survey and provided with a $10 (CAD) honorarium.

### Sample

The sample frame for the study was drawn from MD Select, the CD ROM version of the Canadian Medical Directory. Physicians from the three practitioner groups of interest (respirologists, family physicians, geriatricians) were identified and assigned a number. Random samples were then drawn and potential respondents were contacted by phone to determine their eligibility for the mail survey. This involved confirming that the potential respondent belonged to the practitioner group of interest, was previously unaware of the decision aid, and had patients for whom the decision aid would potentially apply.

A sample size of 450 (150 from each physician group) would have allowed us to estimate the percent indicating an intention to use the decision aid well within a 10% bound on the error of estimation, with 95% confidence. Thus, allowing for a conservative response rate of 60%, a sample frame of 765 (255 practitioners from each physician group) was required. A questionnaire was mailed to random samples of respirologists (n = 255) and family physicians (n = 255). Since there were only 130 geriatricians, the entire eligible population of geriatricians (n = 130) was contacted.

### Questionnaire

Using Likert-scaled items, the two-page survey elicited physicians' perceptions of the characteristics of the decision aid and their willingness to use it. The items were based on: a) the conceptual framework of the study, the Ottawa Model of Research Use[11,12] ; b) the work of Rogers[13], Grilli and Lomas[14], Grol et al[15], and Brouwers et al[16] who have identified attributes or characteristics of innovations related to adoption; and c) a qualitative study we undertook to elicit physicians perceptions of decision aids[3]. The 43 items assessing the characteristics of the decision aid were divided into four main areas related to: the development of the decision aid (4 items); the content and format of the decision aid (10 items); the decision aid meeting patients' needs (11 items); and, the physicians' clinical practice (18 items). The characteristics were rated on a 5-point scale ranging from 1 ('strongly agree') to 5 ('strongly disagree'). Willingness to use the decision aid was assessed by asking the physicians how comfortable they would be to use the decision aid with patients ('very uncomfortable', 'uncomfortable', 'neutral', 'comfortable', or 'very comfortable') and how likely they were to use it within the next three months ('not at all', 'very unlikely', 'somewhat likely', 'likely', or 'very likely'). Additionally, physicians were asked whether they perceived a need for a decision aid on the topic of the particular decision aid they were asked to review. Lastly, physicians were asked the number of years that they had been practicing in their specialty (all other demographic information was obtained from the Canadian Medical Directory).

The follow-up survey inquired whether the physician had carried through with their intention to use the decision aid. Those who responded 'yes' were asked with how many patients they had used the decision aid and whether the experience was positive, negative or neutral. Those who responded 'no' were asked whether they had considered using the decision aid with at least one patient and whether they were considering using it in the future.

Ethical approval for the project was obtained from the Ottawa Health Research Institute Research Ethics Board. Consent to participate in the study was assumed by the respondent returning the completed mail survey and responding to the follow-up survey.

### Data management and analysis methods

All statistical analyses were conducted using SPSS 13.0. A descriptive profile of the professional characteristics of survey responders was generated and the original data set was assessed for missing data. Respondents with two or less missing data points (n = 44) had values imputed based on their respective variable mode and respondents presenting with more than two missing data points (n = 7) were removed from further analyses.

A criterion was established for participation in the follow-up survey. A participant's response to the intention question on how likely they were to use the decision aid was dichotomized into those who were 'likely' or 'very likely' versus all other responses. Only the former group was contacted for the follow-up survey. Two additional items were dichotomized: the comfort item 'How comfortable would you be offering the decision aid to your patients?' was dichotomized into those that were 'comfortable' and 'very comfortable' versus 'neutral', 'uncomfortable', and 'very uncomfortable'; and the question of whether there was need for a decision aid on the topic was dichotomized into those who felt there was a need versus those who were unsure or did not feel there was a need.

Principal components analysis with Verimax rotation was used to reduce the 43 'characteristic' Likert-scaled items to a parsimonious set of meaningful components – which were then used as independent variables in the subsequent logistic regression analysis (the mean score of all items within each extracted component was used as the composite score for each factor). Components were assigned descriptive labels that represented the items within them: Quality and Value for Patients, Value for Physicians, Decision Aid Content, and Implementation issues. Items which loaded upon multiple components were assigned to the component which made most contextual sense. Items which loaded upon multiple components and were not easily assigned to any one of them (n = 1), and items with loadings below 0.45 (n = 3) were removed from further analysis[17].

Logistic regression was used to examine potential factors influencing a practitioners' intention to use the decision aid. *Intention *was used as the dependent variable. Predictors included the dichotomized *comfort *and *need *items and the four components extracted in the principal components analysis. Given potential differences between groups, dummy variables for respirologists and family physicians (with geriatricians as the reference group) were created and included as predictors in the model. Additional demographic variables included: physician's sex, number of years in specialty, having a hospital appointment, and having a university appointment. All predictor variables that were significant at the p = 0.10 level in preliminary univariable analysis were entered into the multivariable model.

## Results

### Physicians' characteristics

Of the 640 surveys mailed out; 60 surveys were returned unopened due to incorrect addresses. Table 1 presents information on study participation and physicians' characteristics. The response rate reflects the number of subjects who agreed to participate in the study, by virtue of completing the survey. Response to the initial mail survey was lower for family physicians (37%) than both geriatricians (58%) and respirologists (50%). The majority of the sample was male and had a hospital appointment. The mean number of years in their specialty varied from 10 years for geriatricians to 18 years for family physicians, with respirologists having a mean of 15 years in their specialty.

**Table 1:** Physician participation and characteristics

	Total	Respirologists	Family Physicians	Geriatricians
Number of MDs contacted	640	255	255	130
Eligible	580	227	231	122
Response rate	270 (47%)	114 (50%)	85 (37%)	71 (58%)
Sex				
Female	79 (29%)	18 (16%)	27 (32%)	34 (48%)
Male	191 (71%)	96 (84%)	58 (68%)	37 (52%)
Mean years in specialty (S.D.)	14.4 (8.1)	14.7 (6.8)	18.0 (9.0)	9.9 (6.9)
Hospital appointment	197 (73%)	96 (84%)	33 (39%)	68 (96%)
University appointment	92 (34%)	50 (44%)	6 (7%)	36 (51%)

### Perceived attributes of decision aids

Table 2 presents the number and percentage of respondents who agreed or strongly agreed with statements describing the development of the decision aid, the content and format of the decision aid, their perceptions of the extent to which the decision aid meets patients' needs, and their perceptions of how the decision aid would influence or impact their clinical practice.

**Table 2:** Physicians' perceptions of characteristics of the patient decision aids (n = 263)

**Characteristic**	Number indicating 4 or 5 on five-point agreement scale*				
	Total	Respirologists (COPD DA)	Family Physicians (HRT DA)	Geriatricians (Tube feeding DA)	Chi-square p-value
**...related to the development of the decision aids**					
Description of risk/benefits of choices supported by evidence	236 (90%)	99 (88%)	80 (95%)	57 (85%)	0.102
Developers credible	233 (89%)	99 (88%)	76 (90%)	58 (87%)	0.752
Decision aid well developed	226 (86%)	88 (79%)	79 (94%)	59 (88%)	0.007
Decision aid not influenced by vested interests	154 (59%)	78 (70%)	35 (42%)	41 (61%)	<0.0001
**...related to the content and format of the decision aids**					
Essential information for decision making provided	239 (91%)	98 (88%)	78 (93%)	63 (94%)	0.254
Well organized	234 (89%)	98 (88%)	79 (94%)	57 (85%)	0.175
Information on choices balanced	230 (87%)	92 (82%)	80 (95%)	58 (87%)	0.023
Evidence about choices presented in unbiased manner	227 (86%)	94 (84%)	74 (88%)	59 (88%)	0.626
Evidence presented reflects my understanding of the data	226 (86%)	92 (82%)	69 (82%)	65 (97%)	0.01
Presents probabilities of risk/benefits in understandable manner	223 (85%)	95 (85%)	70 (83%)	58 (87%)	0.86
Clearly describes treatment choices	221 (84%)	94 (84%)	74 (88%)	53 (79%)	0.325
Worksheet adds value to decision aid	202 (77%)	85 (76%)	66 (79%)	51 (76%)	0.897
Evidence presented is up-to-date	197 (75%)	88 (79%)	47 (56%)	62 (93%)	<0.0001
Combination of workbook and tape good	196 (75%)	86 (77%)	63 (75%)	47 (70%)	0.61
**... related to the decision aid meeting patients' needs**					
Helps patients understand risk/benefits of treatment choices	228 (87%)	95 (85%)	73 (87%)	60 (90%)	0.664
Allows patients to participate as they wish in decision making process	217 (83%)	92 (82%)	73 (87%)	52 (78%)	0.325
Guides patients through decision making in logical fashion	216 (82%)	93 (83%)	68 (81%)	55 (82%)	0.931
Helps prepare patients for decision making	216 (82%)	89 (79%)	71 (85%)	56 (84%)	0.617
Will be acceptable to patients	214 (81%)	84 (75%)	71 (85%)	59 (88%)	0.063
Helps patients in reaching a decision	211 (80%)	87 (78%)	71 (85%)	53 (79%)	0.475
Will improve patients' decision making	207 (79%)	88 (79%)	70 (83%)	49 (73%)	0.314
Will produce greater good than harm	204 (78%)	88 (79%)	62 (74%)	54 (81%)	0.577
Apply to sizable proportion of patients	178 (68%)	81 (72%)	54 (64%)	43 (64%)	0.383
Will be simple to use	168 (64%)	77 (69%)	51 (61%)	40 (60%)	0.364
Decision aid provides information that is not too complex for patients	143 (54%)	73 (65%)	36 (43%)	34 (51%)	0.006
**... related to physicians' clinical practice**					
Compatible with how I think patients should be informed about choices	219 (83%)	90 (80%)	71 (85%)	58 (87%)	0.522
Will complement my usual approach	208 (79%)	88 (79%)	64 (76%)	56 (84%)	0.532
Will be useful in my practice	188 (71%)	79 (71%)	58 (69%)	51 (76%)	0.606
Will improve my usual approach	179 (68%)	77 (69%)	58 (69%)	44 (66%)	0.888
Reliable tool for helping patients	173 (66%)	64 (57%)	67 (80%)	42 (63%)	0.004
Will help me understand issues important to patient	167 (63%)	80 (71%)	52 (62%)	35 (52%)	0.033
Will help me tailor counselling to patients' needs	162 (62%)	74 (66%)	50 (60%)	38 (57%)	0.412
Will be easy to use in my practice	160 (61%)	66 (59%)	48 (57%)	46 (69%)	0.305
Will positively affect my relationship with patients	154 (59%)	58 (52%)	55 (65%)	41 (61%)	0.138
Will not require major changes	152 (58%)	61 (54%)	44 (52%)	47 (70%)	0.058
Easy to experiment with before deciding to adopt	142 (54%)	52 (46%)	45 (54%)	45 (67%)	0.026
Will improve quality of patient visits	140 (53%)	56 (50%)	53 (63%)	31 (46%)	0.08
Will increase patient satisfaction with my care	140 (53%)	50 (45%)	55 (65%)	35 (52%)	0.015
Will not require reorganization of my practice	129 (49%)	55 (49%)	36 (43%)	38 (57%)	0.239
Would not be used if I am required to purchase it	122 (46%)	39 (35%)	58 (69%)	25 (37%)	<0.0001
Will provide easily observable benefits for the patient	98 (37%)	37 (33%)	38 (45%)	23 (34%)	0.184
Likely to be used by most of my colleagues	85 (32%)	43 (38%)	17 (20%)	25 (37%)	0.016
Will save me time	70 (27%)	22 (20%)	27 (32%)	21 (31%)	0.088

*On scale: 1 indicates strongly disagree; 5 indicates strongly agree

Abbreviations: COPD = chronic obstructive pulmonary disease; DA = decision aid; HRT = hormone replacement therapy

More than 85% of respondents felt the decision aid they reviewed was well developed by credible developers and that the presentation of risks and benefits was supported by evidence. However, only 59% felt the decision aid was not influenced by vested interests. There were two differences by physician group: respirologists were less likely to agree that the COPD decision aid was well developed; and fewer family physicians felt the hormone replacement therapy decision aid was free of the influence of vested interests.

More than 85% of the respondents felt the decision aid presented the essential information for decision making in an organized, balanced, understandable and unbiased manner. While the majority of all physician groups (86%) felt that the information presented reflected their understanding of the data, more geriatricians (97%) agreed with this characteristic. The majority of respondents (≥75%) also felt that the decision aid clearly described the treatment choices, had an appropriate format, and was based on up-to-date evidence. For the latter characteristic, there was some difference by specialty and only 56% of family physicians felt that the decision aid presented up-to-date evidence.

A majority of respondents (≥80%) felt that the decision aid would help patients understand the benefits and risks of the options; that it would guide patients through the decision making process in an acceptable and logical way; and that it would help them to prepare for and reach a decision. More than 60% of respondents felt that the decision aid would improve patients' decision making, would be beneficial and simple to use, and that it would apply to a large proportion of patients. Fewer respondents (54%) felt that the decision aid provided information that was not too complex for patients.

The majority of respondents(>60%) felt that the decision aid was compatible with their view of how patients should be informed about choices and that use of the decision aid would improve their usual approach as well as help them to tailor their counselling to patients' needs. However, overall they were less inclined to believe that it would improve the quality of patient visits or increase patient satisfaction with care (53%). More family physicians indicated that they would not use the decision aid in their practice if they were required to incur the expense of purchasing it and they were also less likely to believe that the decision aid would be used by their colleagues. Overall, very few respondents (27%) felt that use of the decision aid would save time.

### Intentions to use the decision aid

Table 3 presents information on physicians' perceptions of the decision aid and intentions to use it. Overall, physicians felt there was a need for a decision aid on the topic they reviewed. However, family physicians were significantly less likely than respirologists or geriatricians to see the need for the decision aid. Sixty-four percent of the family physicians felt there was a need for a decision aid for post-menopausal women considering long-term hormone replacement therapy, 84% of the geriatricians felt there was a need for a decision aid for individuals faced with making a decision about long-term feeding tube placement in a cognitively impaired older person, and 86% of the respirologists felt there was a need for a decision aid for individuals with COPD considering mechanical ventilation at the end of their lives. Eighty-one percent of the physicians surveyed felt 'comfortable' or 'very comfortable' with offering the decision aid to their patients. One-hundred forty-one (54%) physicians indicated that they would 'likely' or 'very likely' use the decision aid in the next three months.

**Table 3:** Physicians' perceptions of and willingness to use patient decision aids (n = 263)

	Total n = 263	Respirologists n = 112	Family Physicians n = 84	Geriatricians n = 67	Chi-square p-value
Need for decision aid	206 (78%)	96 (86%)	54 (64%)	56 (84%)	0.001
Comfortable, very comfortable offering decision aid to patients	212 (81%)	87 (78%)	68 (81%)	57 (85%)	0.478
Likely, very likely to use decision aid within 3 months	141 (54%)	60 (54%)	51 (61%)	30 (45%)	0.149

### Factors influencing intention to use the decision aids

Results of the principal components analysis are presented in Table 4. Twelve characteristics related to decision aid quality and value for patients loaded on to Component 1; 10 characteristics related the decision aids value for physicians loaded on to Component 2; 11 characteristics related to decision aid content loaded on to Component 3; and, 6 characteristics related to implementation of the decision aid loaded on to Component 4. The four characteristics that were removed from the analysis due to low or multiple components loadings are also presented.

**Table 4:** Results of principal components analysis

	Component Loading
Component 1 – Quality and Value for Patients	
Helps patients in reaching a decision	0.72
Helps prepare patients for decision making	0.72
Will improve patients' decision making	0.67
Allows patients to participate as they wish in decision making process	0.65
Helps patients understand risk/benefits of treatment choices	0.62
Will be simple to use	0.59
Apply to sizable proportion of patients	0.59
Guides patients through decision making in logical fashion	0.59
Will be acceptable to patients	0.53
Well organized	0.50
Presents probabilities of risk/benefits in understandable manner	0.49
Combination of workbook and tape good	0.49
Component 2 – Value for Physicians	
Will improve quality of patient visits	0.79
Will increase patient satisfaction with my care	0.77
Will positively affect my relationship with patients	0.74
Will help me tailor counselling to patients' needs	0.74
Will provide easily observable benefits for the patient	0.71
Will help me understand issues important to patient	0.65
Will save me time	0.63
Will improve my usual approach	0.60
Reliable tool for helping patients	0.50
Will complement my usual approach	0.47
Component 3 – Decision Aid Content	
Evidence presented reflects my understanding of the data	0.72
Evidence about choices presented in unbiased manner	0.71
Information on choices balanced	0.67
Description of risk/benefits of choices supported by evidence	0.66
Decision aid well developed	0.65
Essential information for decision making provided	0.64
Developers credible	0.62
Evidence presented is up-to-date	0.58
Clearly describes treatment choices	0.55
Compatible with how I think patients should be informed about choices	0.51
Decision aid not influenced by vested interests	0.46
Component 4 – Implementation	
Will not require reorganization of my practice	0.73
Will be easy to use in my practice	0.60
Decision aid provides information that is not too complex for patients	0.59
Will not require major changes to the way I currently discuss the topic	0.52
Likely to be used by most of my colleagues	0.47
Easy to experiment with before deciding to adopt it in practice	0.46
**Items removed from factor analysis due to multiple loading or factor loading < 0.45**	
Worksheet adds value to decision aid	<0.45
Will produce greater good than harm	<0.45
Will be useful in my practice	Component 1 and 2
Would not be used if I am required to purchase it	<0.45

Results of the logistic regression model are presented in Table 5 – four variables (sex, years of specialty, hospital appointment, and university appointment) that were not significant at the p = 0.10 level, in the univariable analysis, were not entered into the multivariable analysis. Physicians who responded that they were comfortable or very comfortable with offering the decision aid to their patients were approximately six times more likely to indicate that they intended to use the decision aid in the next three months compared to those who were not comfortable. Physicians who felt that there was a need for a decision aid on the topic they reviewed were three times more likely to intend to use the decision aid. Family physicians were four times more likely to report intent to use the hormone replacement therapy decision aid than geriatricians were to indicate intent to use the long-term placement of a feeding tube decision aid. Respirologists' intentions to use the COPD decision aid were two times more likely than geriatricians' intentions to use to the tube feeding decision aid. Finally, physicians who responded positively towards the ease in which the decision aid could be implemented in their practice had twice the odds of intending to use the decision aid. Factors related to content, the ability of the decision aid to meet patient needs, and the value for physicians were not significantly associated with intent to use the decision aid.

**Table 5:** Results of the logistic regression model used to predict intention to use the patient decision aid (n = 263)

Predictor	Intention		Odds ratio (95% confidence interval)	P-value
	No n = 122	Yes n = 141		
**Comfort with offering patient decision aid to patients**				<0.0001
Very uncomfortable/uncomfortable/neutral (ref)	44	7		
Comfortable/very comfortable	78	134	5.7 (2.2 – 14.8)	
**Need for a decision aid on the topic**				0.005
No/unsure (ref)	40	17		
Yes	82	124	3.1 (1.4 – 6.8)	
**Decision Aid**				0.004
Tube feeding (ref)	37	30		
Hormone replacement therapy	33	51	4.2 (1.8 – 9.8)	
COPD	52	60	2.3 (1.1 – 4.8)	
**Patient decision aid characteristics**				
Implementation			2.4 (1.3 – 4.4)	0.006
Value for Physicians			1.6 (0.8 – 3.1)	0.206
Decision Aid Content			1.4 (0.6 – 3.1)	0.433
Quality and Value for Patients			1.0 (0.4 – 2.5)	0.932

Abbreviations: COPD = chronic obstructive pulmonary disease; ref = reference group

### Use of the patient decision aids

One-hundred forty-one (54%) physicians indicated that they would 'likely' or 'very likely' use the decision aid in the next three months. However, of those who responded to the three month follow-up telephone survey (n = 99), only 32% had followed through with this intention (Table 6).

**Table 6:** Physicians' use of the patient decision aids (n = 263)

	Total n = 263	Respirologists n = 112	Family Physicians n = 84	Geriatricians n = 67	Chi-square p-value
Likely, very likely to use decision aid within 3 months	141 (54%)	60 (54%)	51 (61%)	30 (45%)	0.149
Response rate	99 (70%)	47 (78%)	31 (61%)	21 (70%)	
Used at 3 months	32 (32%)	12 (26%)	16 (52%)	4 (19%)	0.02

## Discussion

This is the first random sample survey to show that physicians from different specialties consider some select decision aids necessary and overwhelmingly see them as high quality products that are useful to patients. Most physicians were comfortable with patients using the decision aids and a majority of physicians considered using them in their practices. Factors that were associated with their intention to use a decision aid related to their comfort level with offering it to patients, the specific decision aid topic (which may be related to opportunity), and concerns about implementing the decision aid in their practice. While more than half the physicians reported they would use the decision aid in the next three months, only a minority of those who stated that they would be likely to use the decision aid actually carried through with this intention.

Our findings support reports in the literature that physicians perceive implementation of shared decision making strategies to be time consuming[2,3,18-28] and potentially lacking applicability to patients due to the complexity of information or patients' characteristics[2,3,18,19,22,26-29]. Contrary to previous research which indicated that physicians perceived patients' preference for decision-making being incompatible with a shared decision making model[2,24,27], the majority of physicians in this survey perceived the decision aid as being an effective method to meet patients decision making needs and compatible with their beliefs of how patients should be informed about choices. Despite strong support for the format, content and quality of the decision aids, this analysis revealed that intention to use the decision aid was more strongly influenced by the logistical issues of implementing the decision aid in the physicians' practice.

There has been a considerable amount of research around facilitators and barriers to implementation of evidence-based clinical practice. While much of this research has centered on the implementation of guidelines, there has been increasing focus on the implementation of shared decision making strategies. A recent review provided an overview of barriers and facilitators to implementing decision aids in clinical practice[4]. The review points out the need to address factors related to: the attributes of decision aids, the practitioners and patients who use them, and the practice environment in which they are used, to successfully implement decision aids into the process of care.

In keeping with the literature, we also found that time and organizational issues can be significant barriers to the implementation of patient decision aids[4,30,31]. Organizational issues include limited space or access to decision aid materials – resulting in diminished referral to the resources [2,30]. Time issues fall into two distinct categories, the time required to access the information and provide it to patients and the time required in consultation to discuss the options[24,25,30,31]. Time constraints in clinical practice pose a formidable obstacle to introducing patient decision aids into the consultation process. However, there is recent evidence to suggest that implementing shared decision making in practice may not require more time than usual care[32]. This suggests that further research into the time required to accommodate patient decision aids into routine care is required as well as research into how other members of the healthcare team may be involved in facilitating the use of decision aids.

Resistance to change has been identified as another barrier to the implementation of patient decision aids and this resistance can come from varied sources including limitations of the evidence, a desire to individualize patient care, and determination to maintain the status quo[4,31]. Physicians may distrust the evidence, question its relevance to individual patients, question its interpretation or feel that there is insufficient evidence[4,25,31]. Physicians may feel that standardizing patient care disrupts the doctor-patient relationship or hinders the consultation process [25,31]. Maintenance of the status quo may derive from the belief that they are already adequately educating patients and involving them in decision making[30]; or it may stem from the preference of a paternalistic role and the genuine belief that the approach is better for patients[30]. These factors were not strongly supported by the study findings.

The main limitations of this study are related to the response rate, reliance on self-reported variables, and possible social response bias. The overall response rate was 47% and ranged from 37% for family physicians to 58% for geriatricians. We have limited data on the characteristics of the physicians to determine whether non-responders differed in some meaningful way from responders. Therefore caution is required when generalizing the results of the study to these physician groups. Caution must also be used in interpreting the findings since a different decision aid was assessed by each specialty. It is not possible to disentangle whether the physicians' perceptions of the attributes of the decision aid were related solely to the decision aid or to characteristics of the physician group assigned each of the decision aids. Another potential source of bias in this study is the reliance on a self-reported variable for use of the decision aid. Studies that have compared self-reported compliance with actual use of practice guidelines found that physicians tended to overestimate their use of the guidelines[14,33,34]. This may mean that there is a discrepancy between the reported and actual use of decision aids in this study. However, the likely tendency would have been for physicians to overestimate their use of the decision aid, meaning that that is a greater gap between intention to use the decision aid and behaviour. Lastly, it is possible that respondents offered more positive responses than they actually believed, providing what they perceived to be socially desirable answers. This social response bias could lead to over-reporting their support for decision aids. The results of this study need to be taken in context of the above mentioned potential sources of bias.

## Conclusion

Improving decisions in health care requires both better informed patients and clinicians working together. Evidence-based patient decision aids are intended to support patients in making informed values-based choices and to supplement the patient-clinician interaction. Despite the fact that physicians perceive patient decision aids to be high quality products that are useful to patients, they have not been widely adopted by physicians.

Physicians' intentions to use decision aids are related to their level of comfort in offering them to their patients, differ by decision aid topic, and are related to physicians' concerns about implementation issues. Our survey also reveals that there is a considerable gap between physicians' intentions to adopt patient decision aids in their clinical practices, and their self-reported behavior. Further research is required to study the determinants of this intention-behaviour gap and to develop interventions aimed specifically at barriers to physicians' use of decision aids. As long as physicians are not using patient decision aids, patients will not be able to benefit from this technology.

## Competing interests

The author(s) declare that they have no competing interests.

## Authors' contributions

IG, JL, AO, JT conceptualized and designed of the study. AO, SLM, PH, SA developed the decision aids used in the survey. SLM, AC, PH, SA advised on physician recruitment strategies. IG, CB, JP, JT participated in analyzing the data and all authors contributed to interpretation of the data. IG, CB developed the first draft of the manuscript. All authors participated in critical revision of the draft manuscript. All authors read and approved the final manuscript.

## Pre-publication history

The pre-publication history for this paper can be accessed here:


